# Validation of Molecular Dynamics Simulations for Prediction of Three-Dimensional Structures of Small Proteins

**DOI:** 10.3390/molecules22101716

**Published:** 2017-10-12

**Authors:** Koichi Kato, Tomoki Nakayoshi, Shuichi Fukuyoshi, Eiji Kurimoto, Akifumi Oda

**Affiliations:** 1 Graduate School of Pharmacy, Meijo University, 150 Yagotoyama, Tempaku-ku, Nagoya, Aichi 468-8503, Japan; 144331503@ccalumni.meijo-u.ac.jp (K.K.); ray-sc76@stu.kanazawa-u.ac.jp (T.N.); kurimoto@meijo-u.ac.jp (E.K.); 2 Department of Pharmacy, Kinjo Gakuin University, 2-1723 Omori, Moriyama-ku, Nagoya, Aichi 463-8521, Japan; 3 Institute of Medical, Pharmaceutical and Health Sciences, Kanazawa University, Kakuma-machi, Kanazawa, Ishikawa 920-1192, Japan; fukuyosi@p.kanazawa-u.ac.jp; 4 Institute for Protein Research, Osaka University, 3-2 Yamadaoka, Suita, Osaka 565-0871, Japan

**Keywords:** molecular dynamics simulation, protein structure prediction, replica exchange molecular dynamics, secondary structure

## Abstract

Although various higher-order protein structure prediction methods have been developed, almost all of them were developed based on the three-dimensional (3D) structure information of known proteins. Here we predicted the short protein structures by molecular dynamics (MD) simulations in which only Newton’s equations of motion were used and 3D structural information of known proteins was not required. To evaluate the ability of MD simulationto predict protein structures, we calculated seven short test protein (10–46 residues) in the denatured state and compared their predicted and experimental structures. The predicted structure for Trp-cage (20 residues) was close to the experimental structure by 200-ns MD simulation. For proteins shorter or longer than Trp-cage, root-mean square deviation values were larger than those for Trp-cage. However, secondary structures could be reproduced by MD simulations for proteins with 10–34 residues. Simulations by replica exchange MD were performed, but the results were similar to those from normal MD simulations. These results suggest that normal MD simulations can roughly predict short protein structures and 200-ns simulations are frequently sufficient for estimating the secondary structures of protein (approximately 20 residues). Structural prediction method using only fundamental physical laws are useful for investigating non-natural proteins, such as primitive proteins and artificial proteins for peptide-based drug delivery systems.

## 1. Introduction

Various methods for precise three-dimensional (3D) protein structure prediction have been developed [1,2,3]. Homology modeling, in which 3D structural models are generated from known experimental homologue protein structures, can provide high precision structural models for drug discovery applications [4,5]. Even when the structure of a homolog is not available, structural modeling is possible using protein threading and ab initio protein modeling [2,6]. Iterative Threading ASSEmbly Refinement (I-TASSER) is one of the most successful protein structure prediction methods [7,8]. This method requires detection of structural templates from the Protein Data Bank (PDB) by threading. In Phyre2, another well-known protein structure prediction method, secondary structures are predicted on the basis of amino acid sequences, and then loops are created to connect the structural motifs [9]. These methods are used for proteins consisting of approximately 20 natural amino acids (Magic 20) because the structural information of existing proteins is used. For 3D structural predictions of non-natural proteins, such as those with amino acid residues other than the Magic 20 and/or very short proteins, these prediction methods frequently fail to construct 3D structures. For example in SWISS-MODEL [10], the target sequence must be >30 residues, and predictions for shorter sequences are not possible. In addition, these structure prediction methods are inappropriate for generating conformer sets for short proteins or peptides because these methods can generate only a limited number of structures. In a typical case, only one structural model is obtained for one input protein sequence. Even if the set of conformations were generated, the conformational tendency based on statistical mechanics ensembles would not be evaluated. For short protein structure predictions, PEP-FOLD is a well-known successful method; however, this approach requires known protein structures for constructing structure fragments and cannot be used for peptides with d-amino acids or unusual amino acids [11,12]. The proteins containing d-amino acids and/or β-amino acids can result in the protein conformational changes and cause aggregation leading to diseases [13,14,15]; therefore, predictions of those structures in the same way as ordinary proteins are inappropriate. In such cases, it is necessary to estimate proteins/peptides structures by methods that do not refer to 3D structures of known proteins consisting of the Magic 20. 3D structure predictions of non-natural proteins are important for artificial protein design for drug design and development. Recently, peptide-based drug delivery systems (DDS) have been developed and methods for directly combining a drug and a peptide, such as peptide drug conjugates, are of particular interest [16]. In particular, peptide-based DDS using cell-penetrating peptides (CPPs) have been developed [17]. Most CPPs have cationic properties, some of which arrange a plurality of basic amino acid residues; however, some form helix structures to concentrate the positive charge by the tertiary structures [17,18]. The development of DDS using such peptides that form specific structures will be improved by structure prediction methods for small proteins and such methods are important for drug development. In addition, non-natural proteins that are not presently found on the Earth frequently play significant roles in investigations of the origin of life. Although extant proteins consist of the Magic 20, many researchers have proposed that the earliest primitive proteins were constructed of a more limited number of amino acids [19,20,21,22,23]. For example, Ikehara et al. [23] hypothesized that life originated from proteins constructed of only glycine, alanine, aspartic acid, and valine. These proteins are called as [GADV]-proteins. Van der Gulik et al. [21] also hypothesized that [GADV]-peptides and metal ions played important roles in the protobionts of the RNA world hypothesis. Structural predictions of these primitive proteins are useful for investigations of the chemical (and biological) environment of primitive Earth.

For protein structure calculations, molecular dynamics (MD) simulations use only Newton’s equations of motion, and no information on the 3D structures of extant proteins is required. Thus, MD simulations seem to be appropriate for structural predictions of non-natural proteins, such as artificial proteins for peptide-based DDS and primitive proteins. Oda et al. described the efficacy of MD simulations in predicting 20 residue [GADV]-protein structures [24], where the secondary structure formation tendencies of the [GADV]-proteins can be clarified using MD simulations. In addition, several properties of the 42 residue amyloid β1–42 containing d-aspartic acid can be estimated by the replica exchange MD (REMD) method [25,26]. Therefore, MD simulations may be effective for predicting short protein structures and for investigation of the conformational tendencies of peptides. In this study, we simulated proteins of 10–46 residues with known 3D structures to investigate whether MD simulation is effective for short-sequence protein structure predictions. These test proteins are shown in Table 1. Chignolin, CLN025, and Trp-cage, comprising 10–20 residues, are often used for folding simulations [27,28,29]. 2I9M is also used as a model of folding because of its rapid folding. The structure is reproduced by the polarization-adapted hydrogen-bond-specific charge scheme; however, it is difficult to simulate using normal MD simulations [30,31]. In full sequence design 1 of the beta alpha motif (FSD-1), the folding process is observed by action-derived molecular dynamics. In contrast, majority conformations observed in normal MD simulations do not converge to the experimental structure [32,33]. Human parathyroid hormone (HPH), which is the extracted 1–34th residue of parathyroid hormone, was used as a model of structure prediction by generalized-ensemble simulations [34,35]. The highly ordered crystal structure of crambin has been solved and is used as a model of small proteins for structure predictions and simulations [36,37,38,39]. In this study, 3D structural predictions of these small proteins by MD simulations were performed using the same conditions. Although several procedures, as described above, have been frequently used in previous studies, in this study, we tested normal MD and REMD simulations without special treatments under consistent conditions. After the simulations, we evaluated whether our results reproduced the experimental structures. We assessed not only convergence to the “correct answer” structure, but also the ability of the method to estimate the approximate shape, i.e., the reproduction of secondary structure formation. The goals of our study were to test the abilities of MD simulations to predict the small protein structures and to clarify the limitations of this approach.

## 2. Results and Discussion

### 2.1. Root Mean Square Deviations (RMSDs) between Predicted and Experimental Structures

**Figure 1:**
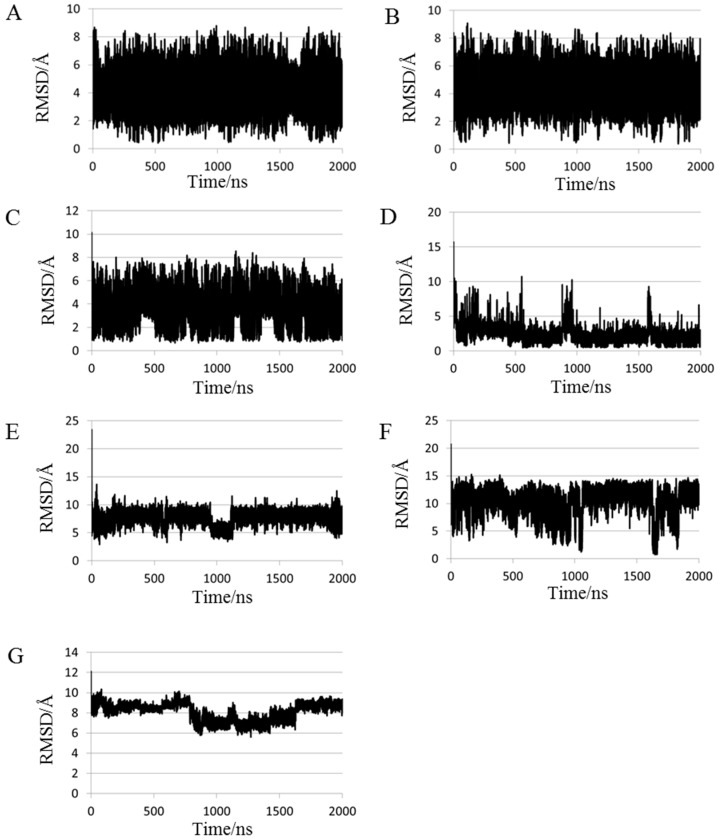
Root mean square deviations (RMSDs) of (**A**) chignolin; (**B**) CLN025; (**C**) 2I9M; (**D**) Trp-cage, (**E**) FSD-1; (**F**) HPH; and (**G**) crambin in normal MD simulations. The reference structures for RMSD calculations were experimental structures shown in Table 1.

**Figure 2:**
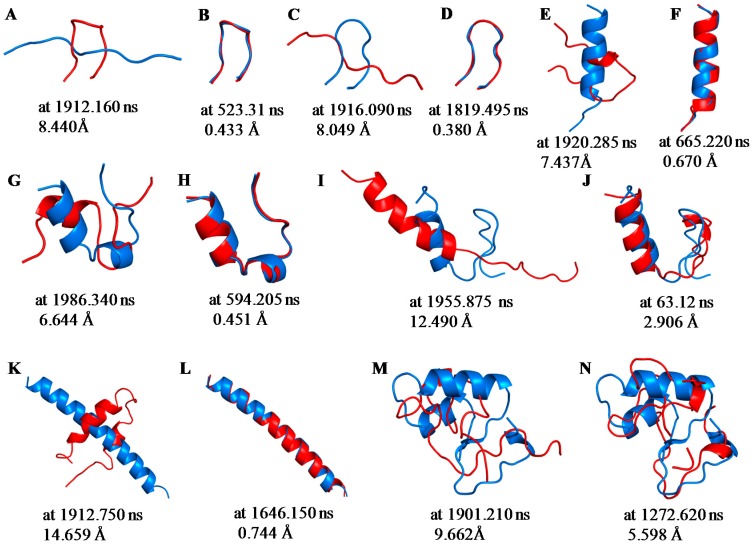
Snapshots at the time of maximum (**A**,**C**,**E**,**G**,**I**,**K**,**M**) and minimum (**B**,**D**,**F**,**H**,**J**,**L**,**N**) RMSD values. The experimentally determined structures (blue) and calculated structures (red) of chignolin (**A**,**B**); CLN025 (**C**,**D**); 2I9M (**E**,**F**); Trp-cage (**G**,**H**); FSD-1 (**I**,**J**); HPH (**K**,**L**) and crambin (**M**,**N**) are illustrated along with the simulation time and the RMSD values.

The smallest root mean square deviation (RMSD) values during MD trajectories compared with experimental structures are shown in Table 2 to evaluate whether short proteins can form 3D-structures in MD simulations. Minimum RMSD values have frequently been discussed in previous studies [27]. As depicted in this table, calculated structures that were close to the experimental structures (RMSD < 2.0 Å) were obtained for chignolin, CLN025, 2I9M and Trp-cage using 200-ns normal MD simulations at 300 K. It is expected that computations of approximately 20 residue proteins can achieve a close match to the experimental structures using 200-ns MD simulations. RMSDs of all proteins, except FSD-1, decreased for 2000-ns MD simulations compared with those obtained using 200-ns MD simulations. Especially, RMSD of both chignolin and HPH were <1.0 Å. Therefore, structures closely approximating the experimental structures can be obtained for 10–34 residue proteins using 2000-ns MD simulations. In contrast, even the minimum RMSD value of crambin is substantial; consequently, it may be difficult to apply this method to >40 residue protein structure predictions. Because Table 2 shows the minimum value of RMSD, the closest structures to experimental data were not obtained in the end of simulations. A previous study reported that the tertiary structure for chignolin and Trp-cage was reproduced by MD simulations; however, the structures closest to the experimental data were not obtained at the end of the simulations [29]. To evaluate convergences of the simulations, RMSD plots are shown in Figure 1 for all simulations, and average RMSD values at the end of simulations are shown in Table 3. For the average calculations, the last 10 ns of the trajectories for 200-ns MD simulations and the last 100 ns of the trajectories for 2000-ns MD simulations were used to eliminate bias from the initial structure. As shown Figure 1, the RMSD values of all proteins largely altered during the simulation and neither predicted structures converged. Even proteins with small minimum RMSD values, as shown in Table 2, did not converge to resemble the experimental structure at the end of the simulation. Among these, the average RMSD value for Trp-cage < 2.0 Å by 2000-ns simulation, and the extension of the simulation time improved the average RMSD of 2I9M. These results indicate that structures closely matching the experimental structures for 20-residue proteins are obtained at the end of 2000-ns MD simulations. In particular, the experimental structure is generally well reproduced for Trp-cage. In chignolin and CLN025, whose minimum RMSD values were small, the average RMSD values were large. The structural fluctuations of these proteins were assumed to be large because of fewer intermolecular interactions. The snapshots of the small test proteins whose RMSDs were the maximum and the minimum values calculated by the 2000-ns MD simulations are shown in Figure 2. The minimum values are shown in Table 2, and the maximum values were evaluated throughout the last 100 ns trajectories. The minimum RMSD structures almost completely matched the experimental structures in chignolin, CLN025, 2I9M, Trp-cage, and HPH. Also in that of FSD-1, the helix-forming region was consistent. In addition, the calculated structure for Trp-cage resembled the experimental structure in the structural motif formation even when RMSD values were maximum (Figure 2G). Also for FSD-1 and HPH, helical tendencies could be reproduced even for the conformations with maximum RMSDs (Figure 2I,K).

### 2.2. Analyses of Secondary Structures

Even if the simulated structures did not converge to match the experimental structures at the end of the simulations, secondary structures may be accurately predicted by MD simulations because secondary structures acquire a rough form prior to folding [29]. Furthermore, some structural motif formations of the maximum RMSD structures were similar to experimental structures, as shown in Figure 2. Therefore, 3D structures of small proteins can be roughly predicted using snapshots as the structural ensemble but not a single snapshot. Even if the fluctuations in structural motifs are large, structure formation tendency can be predicted by accurate assignments of secondary structures.

**Figure 3:**
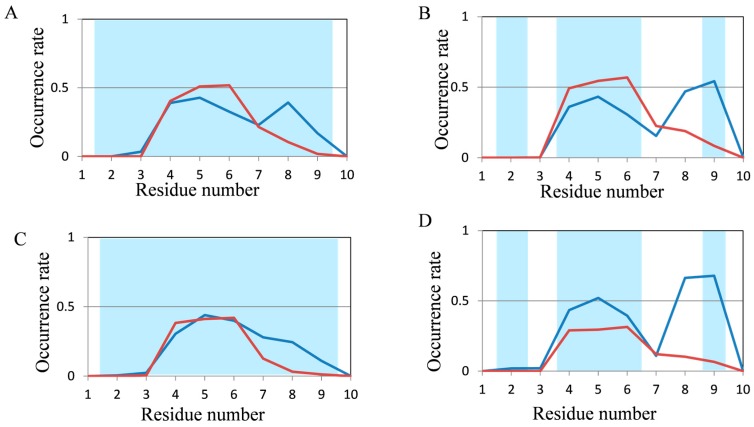
Analysis of the secondary structure formation in chignolin and CLN025 (10 residues) by normal MD simulations. The occurrence rate of secondary structures of (**A**) chignolin; and (**B**) CLN025 in 200-ns simulations and (**C**) chignolin; (**D**) CLN025 in 2000-ns simulations. Red and blue lines show the occurrence rate of helix and β structures in MD simulations, respectively. The background of the graph area for the residues forming helix and β structures in the experimental structures are colored in light red and light blue, respectively.

To evaluate the abilities of MD simulations, we evaluated the occurrence frequencies of secondary structures (helix and β structures) in the last 10 ns and the last 100 ns of trajectories for 200-ns and 2000-ns normal MD simulations, respectively. In order to follow protein folding, the secondary structure formation process is important; however, we investigated not protein folding but protein structure prediction in this study. Thus, we focused on the evaluation of secondary structure tendencies, and the snapshots were used not as time-course data but as ensembles. Occurrence rates of secondary structures in chignolin and CLN025, both of which comprise 10 amino acid residues, are shown in Figure 3. The red and blue lines show the occurrence rate of helix and β structures in MD simulations, respectively. The background of the graph area for the residues forming helix and β structures in the experimental structures are colored in light red and light blue, respectively.

**Figure 4:**
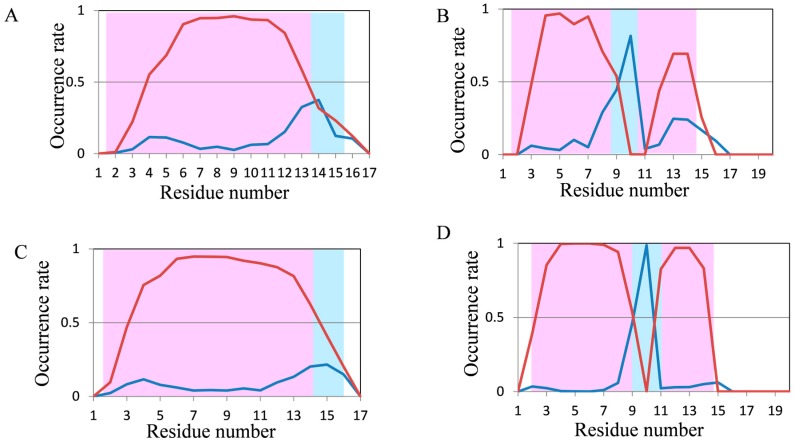
Analysis of the secondary structure formation in 2I9M (17 residues) and Trp-cage (20 residues) using normal MD simulations. The occurrence rate of secondary structures of (**A**) 2I9M; (**B**) Trp-cage using 200-ns simulations and (**C**) 2I9M and (**D**) Trp-cage in 2000-ns simulations. Color coding is the same as Figure 3.

In both proteins, the experimental structures were reproduced in some residues; however, the occurrence rates of secondary structures were generally low. In CLN025, the structure was comparatively close to the experimental data because the occurrence rate of β structures was greater than that of helix structures obtained by 2000-ns MD simulations. The results for 2I9M (17 residues) and Trp-cage (20 residues) are shown in Figure 4. The experimental secondary structures were sufficiently reproduced using 200-ns MD simulations for these proteins. In 2I9M, although the occurrence rate of β structures in 15th residue was lower than that of helix structures, the 200-ns MD simulation reproduced the experimental secondary structures for all other residues (Figure 4A). The secondary structures of Trp-cage were reproduced in all residues using 200-ns MD simulations, furthermore, the differences in occurrence rates between helix and β structures became clearer by 2000-ns MD simulation (Figure 4B,D). These results suggest that secondary structures in for approximately 20-residue proteins can be reproduced using normal MD simulations.

**Figure 5:**
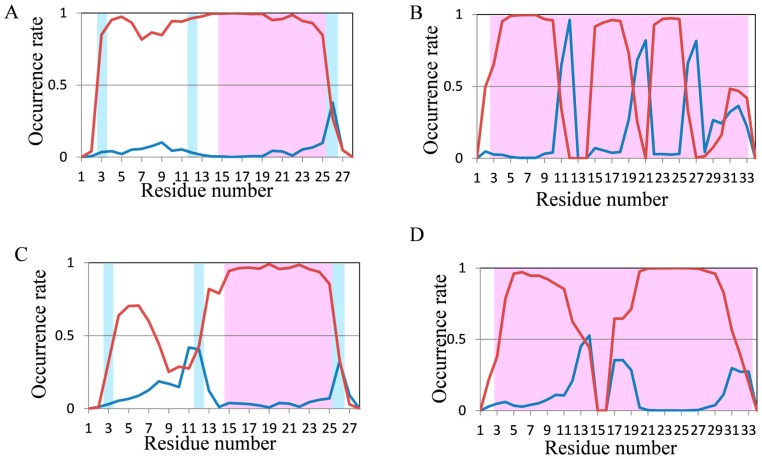
Analysis of the secondary structure formation in FSD-1 (28 residues) and HPH (34 residues) by normal MD simulations. The occurrence rate of secondary structures of (**A**) FSD-1; (**B**) HPH in 200-ns simulations and of (**C**) FSD-1, and (**D**) HPH in 2000-ns simulations. Color coding is the same as that in Figure 3.

**Figure 6:**
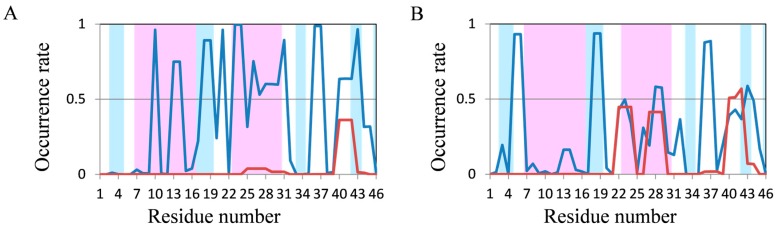
Analysis of the secondary structure formation in crambin (46 residues) using normal MD simulations. The occurrence rate of secondary structures of crambin using 200-ns simulations (**A**) and 2000-ns simulations (**B**). Color coding is the same as that in Figure 3.

In 200-ns MD simulations of FSD-1 and HPH (Figure 5A,B), which are longer proteins, high occurrence rates of secondary structures were observed; however, these occurrence rates were not consistent with the experimental structures included in the Protein Data Bank (PDB). The N-terminus of FSD-1 forms a loop structure in the experimental data, whereas helices were formed in most of the residues by 200-ns MD simulation (Figure 5A). On the other hand, the occurrence rate of helix structures in the N-terminus of FSD-1 was lower in the 2000-ns MD simulation, which was relatively close to the experimental structure (Figure 5C). In a previous study on the folding process of FSD-1 by Lee et al. [32], it was reported that FSD-1 forms β structures after forming helix structures. Because the occurrence rates of the helix structures decreased in our 2000-ns simulations compared with those in our 200-ns simulations, the results shown in Figure 5A,C were consistent with the presumptions proposed by Lee et al. The extension of the simulation time remarkably improved the occurrence rate of secondary structures in HPH and the experimental secondary structure was almost reproduced, except that the occurrence rate of helix structures decreased at the 15–17th residues (Figure 5B,D). With respect to crambin, the secondary structure was not consistent with the experimental data in 200-ns MD simulation (Figure 6). It was somewhat improved by 2000-ns MD simulation, but it could still not explain the experimental structures.

### 2.3. Structure Predictions Using REMD

**Figure 7:**
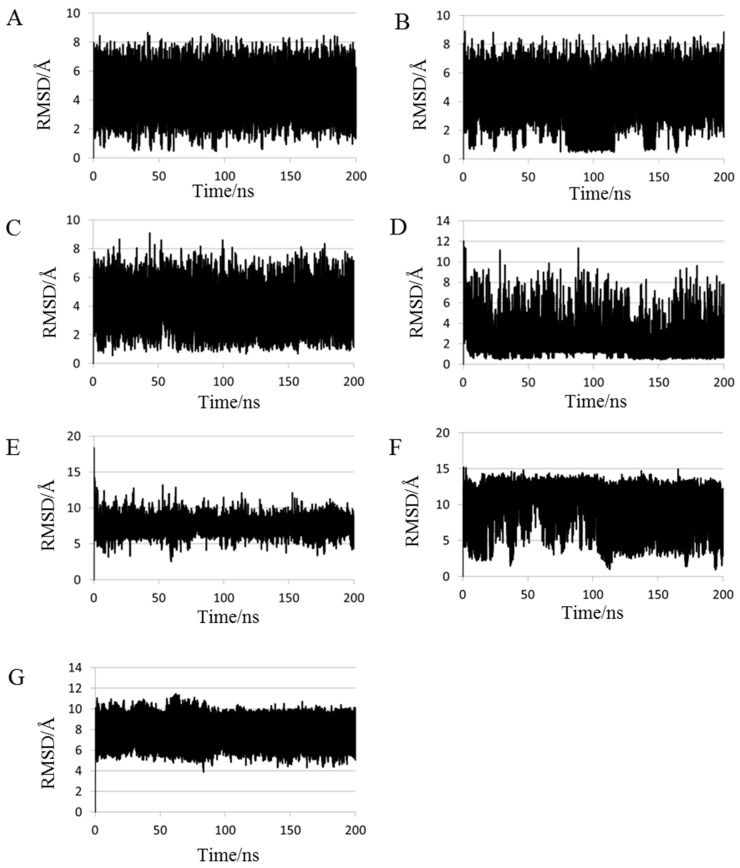
Root mean square deviations (RMSDs) of (**A**) chignolin; (**B**) CLN025; (**C**) 2I9M; (**D**) Trp-cage; (**E**) FSD-1; (**F**) HPH and (**G**) crambin in REMD simulations. The reference structures of RMSD calculations were experimental structures shown in Table 1.

As mentioned above, structural predictions were performed using normal MD simulations and our results indicated that the secondary structures of proteins composed of <34 residues could almost be predicted. In addition, we investigated whether the “correct answer” was obtained using REMD, which is one of the extended ensemble methods. The RMSD plots obtained using REMD simulations are shown in Figure 7. Large structural fluctuations were observed, and those predicted structures did not converge just like the results from normal MD simulation. The smallest and average RMSDs values between experimental and calculated structures in REMD trajectories are shown in Table 4. The last 10 ns of REMD trajectories were used for RMSD calculations. 

The minimum value of crambin was improved compared with that from normal MD simulations; however, values of the others were comparable to those using 2000-ns MD simulations. The average value of Trp-cage was <2.0 Å; however, it was inferior to that of 2000-ns normal MD simulation. Even though the average value of HPH using REMD was lower than that by normal MD simulations, it was still high at 8.769 Å. As with the results of RMSD analyses, there was little improvement compared with normal MD simulations.

**Figure 8:**
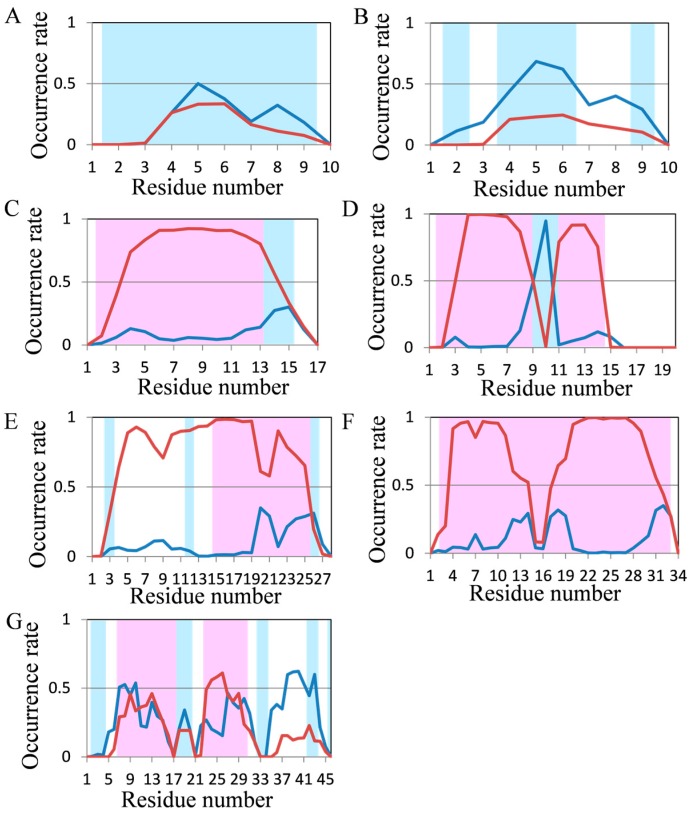
Analysis of the secondary structure formation in seven short proteins by REMD. The occurrence rate of secondary structures of (**A**) chignolin; (**B**) CLN025; (**C**) 2I9M; (**D**) Trp-cage; (**E**) FSD-1; (**F**) HPH and (**G**) crambin in REMD. Color coding is the same as that in Figure 3.

Occurrence rates of secondary structures in the last 5 ns of REMD trajectories were analyzed and the results are shown in Figure 8. In chignolin, the β structure predominated after the 4th residue, although the frequency of secondary structure was comparatively low because of its small size (Figure 8A). The frequency of β structures in CLN025 increased compared with normal MD simulations, but the frequency of the 9th residue was lower than that obtained using 2000-ns MD simulation (Figure 8B). The secondary structures of 2I9M and Trp-cage which were almost reproduced using normal MD simulations, were also reproduced by REMD (Figure 8C,D). The occurrence rates of FSD-1 by REMD were similar to those obtained using 200-ns normal MD simulation, and were worse than those obtained by 2000-ns normal MD simulations (Figure 8E). With respect to HPH, frequency of secondary structures decreased at the 15th to 17th residues; however, predicted structures mostly formed helical structures (Figure 8F).

Similar results were also obtained by 2000-ns normal MD simulations. Compared with structures obtained using normal MD simulations, the predicted structure of crambin was closer to the experimental structure; however, it was far from the “correct answer” (Figure 8G). Because predicted secondary structures using REMD were not significantly close to experimental structures compared with predicted secondary strucures by 2000-ns normal MD simulations for all seven test proteins and because REMD is more time-consuming than normal MD simulations, normal MD simulations are sufficiently accurate for approximately 20 residue protein structure predictions.

### 2.4. Structure Prediction for Primitive Protein

**Figure 9:**
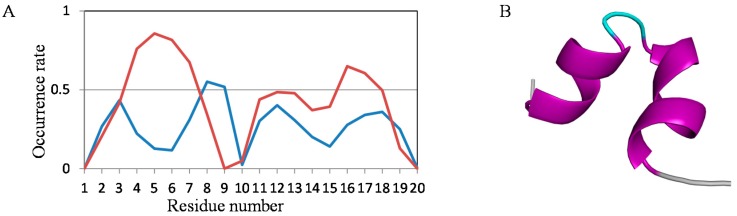
The structure prediction of [GADV]-protein. (**A**) The occurrence rates of secondary structures. Color coding of lines is the same as that in Figure 3; (**B**) The final predicted structure formed at 100 ns. Purple shows helix and light blue shows loop.

It was shown that structure prediction using MD simulation is useful for investigating the tendency of structure formation in short proteins. As a test of this method, the results of simulations for structure prediction of a randomly generated tentative primitive protein constructed of only glycine, alanine, aspartic acid, and valine [GADV]-proteins were determined. The protein has a possibility to form a different structure from all known proteins; therefore, this is a suitable sample for structure prediction using MD simulation. We show an example of the obtained conformation and the occurrence rate of secondary structure using normal MD simulation for one of the randomly generated [GADV]-proteins in Figure 9. The peptide sequence of this test peptide is AADVVAAAAVDVAVGVVVGA. The occurrence rates of helix and β structures for each residue in this peptide are shown (Figure 9A). The frequency of helix structure is high in both the N- and C-terminal regions. Furthermore, Val4–Ala8 and Asp11–Val18 of the final predicted structure (at 100 ns) form helices (Figure 9B). These results are consistent with those obtained using REMD simulation mentioned previously [24]. This test peptide is the “peptide 4” of [24], and the results shown in Figure 9 are similar to those of “peptide 4” in that reference. This result indicates that normal MD simulation is expected to be a useful structure prediction method for short proteins with unknown and non-natural sequences or structures, as well as REMD simulations.

## 3. Methods

The proteins used in this study are shown in Table 1. Linear structures obtained by tleap of AmberTools 12 (University of California, San Francisco, CA, USA) were used for initial structures to eliminate the influence of initial structures as much as possible. Experimental 3D structures used as “correct answer” were downloaded from PDB, except CLN025. The “Correct answer” structure of CLN025 was obtained from [40], because it is not registered in the PDB. For small protein structure predictions, normal MD simulations at a temperature of 300 K and REMD were performed. In normal MD simulations, after 200 cycles of structure optimization, MD simulations of 200-ns and 2000-ns were performed at time step 1 fs. In REMD, eight replicas at the temperatures of 269.5 K, 300.0 K, 334.0 K, 371.8 K, 413.9 K, 460.7 K, 512.9 K, and 570.9 K for chignolin and CLN025; 16 replicas at the temperatures of 169.7 K, 284.4 K, 300.0 K, 316.4 K, 333.8 K, 352.0 K, 371.3 K, 391.7 K, 413.1 K, 435.7 K, 459.6 K, 484.8 K, 511.3 K, 539.3 K, 568.8 K, and 600.0 K for 2I9M, Trp-cage, FSD- 1, and HPH; and 32 replicas at the temperatures of 278.5 K, 285.5 K, 292.7 K, 300.0 K, 307.5 K, 315.2 K, 323.1 K, 331.2 K, 339.5 K, 348.0 K, 356.8 K, 365.7 K, 374.9 K, 384.3 K, 393.9 K, 403.8 K, 413.9 K, 424.3 K, 434.9 K, 445.8 K, 457.0 K, 468.4 K, 480.2 K, 492.2 K, 504.5 K, 517.2 K, 530.1 K, 543.4 K, 557.1 K, 571.0 K, 585.3 K, and 600.0 K for crambin were used. After the structure optimization of 2000 cycles, MD simulation for equilibration at each temperature was performed and then REMD was performed. For equilibration, 200-ps MD simulations were conducted using the 2 fs time step. For REMD, total 200-ns simulations were performed. The time step was 2 fs and the replica was exchanged every 1 ps in REMD. In both normal MD and REMD simulations, we applied SHAKE to constrain the lengths of bonds containing hydrogen atoms [41] and structural restriction was adopted to avoid chiral inversions. In addition, simulations were performed with a salt concentration of 0.1 M in a continuous solvent using GBneck 2 model [42] with mbondi3 atomic radius. For the classical force field, AMBER ff12SB force field was used [43]. No cutoff was used for non-bonding terms of classical molecular mechanical calculations. AMBER 12 was used for all classical MD simulations [44]. The cpptraj module of AmberTools 12 was used for analyses of those results. For both normal MD and REMD simulations, we calculated RMSDs between calculated and experimental structures and evaluated whether experimental structures were reproduced. The snapshots were extracted every 5 ps for normal MD trajectories, and 1 ps for REMD trajectories in RMSD calculations. The secondary structure was identified by the DSSP method [45] for the last 10 ns of the trajectories obtained by 200-ns normal MD and REMD simulations, and for the last 100 ns of the trajectories obtained by 2000-ns normal MD simulations. Then, the occurrence rates of the secondary structures were evaluated through the last 10 ns (200-ns simulations) or the last 100 ns (2000-ns simulations) of the simulations. For example in 200-ns MD simulations, when the occurrence rate of the helix structure was 0.5 for one residue, the residue was included in the helix structures for 5 ns of the last 10 ns of the simulation. The snapshots were extracted every 0.5 ps for normal MD simulations and every 1 ps for REMD. We defined parallel β sheet, antiparallel β sheet and turn as β structures, and α helix, π helix and 3–10 helix as helical structures. The definitions of these secondary structures are identical to those used in previous studies [24,25,46]. For [GADV]-proteins, 100-ns normal MD simulation was performed under similar conditions on seven short proteins, and the last 10 ns trajectory was used for analyses.

## 4. Conclusions

In the present study, we predicted the 3D structures of some proteins with 10–46 residues using MD simulations. Our results suggest that the secondary structures of small proteins are reproduced using normal MD simulations. In particular, the normal MD simulation of Trp-cage showed small average RMSD values and also reproduced the secondary structure; therefore, it was considered that at least the secondary structures of approximately 20 residue protein structures can be accurately predicted by normal MD simulations. In chignolin and CLN025, which have 10 residues and are the smallest of all known proteins, occurrence rates of secondary structures were low and the reproduction of experimental structures was difficult. The reason for this is presumed to be that these proteins are too short to predict folding in MD simulations with an implicit solvent model. This result suggests that a certain length of amino acid sequences and a certain amount of interactions are necessary for protein structure prediction. On the other hand, the structural prediction of crambin was very difficult. For large proteins, it is considered that the folding is practically difficult to simulate using MD simulation with realistic computational time because of extremely large number of degrees of freedom. Most cellular proteins fold on timescales of milliseconds to seconds and several small proteins have been experimentally designed and characterized to fold on a timescale of <20 µs [47]. In this study, we showed the possibility of predicting secondary structures by practically short time simulations. For short protein structure predictions, PEP-FOLD is very effective, especially in producing highly accurate models for proteins of approximately 10 residues [48]. Nevertheless, the accuracy decreases for proteins >20 residues [12,48]. Although our approach is poor for structure prediction of proteins larger or smaller than 20 residues, it has shown good performance for proteins of approximately 20 residues. Furthermore, this can be applied to non-natural sequence proteins. We are working on the development of a biosensor based on coiled-coil [49], and this method may be applicable to the design. This method is sufficiently useful for the investigation of non-natural proteins, such as primitive proteins and artificial proteins for peptide based DDS. We calculated with the implicit solvent model; in contrast, structure prediction of chignolin using REMD simulation with an explicit solvent model has been reported, and the explicit solvent model may decrease fluctuation [50]. However, our research indicates that the tendency of structure formation can be predicted using normal MD simulation with implicit solvent models and can reduce computational costs. In addition, the results for the structural predictions of the proteins other than chignolin, and the predictive ability for secondary structure tendency of normal MD simulations were revealed in this study. Therefore, our study indicates that normal MD simulation is useful for structure prediction for the development of peptides which contain various amino acids and form various structures. In a future study we would like to examine this method on a larger scale with multiple simulation replications.

## Figures and Tables

**Table 1:** Proteins used in this study.

Name	Number of Residues	PDB ID
Chignolin	10	1UAO
CLN025	10	- ^c^
2I9M	17	2I9M
Trp-cage	20	1L2Y
FSD-1 ^a^	28	1FSD
HPH ^b^	34	1ET1
Crambin	46	1CRN

^a^ FSD-1: Full sequence design 1 of the beta alpha motif. ^b^ HPH: Human parathyroid hormone. ^c^ The experimental 3D structure of CLN025 was not in the Protein Data Bank (PDB), but has been reported in [40].

**Table 2:** The minimum RMSDs/Å values between calculated and experimental structures during MD trajectories.

Protein	200 ns	2000 ns
Chignolin	1.338	0.433
CLN025	0.501	0.380
2I9M	0.791	0.670
Trp-cage	0.747	0.451
FSD-1	2.906	2.906
HPH	3.258	0.744
Crambin	7.496	5.598

**Table 3:** Average RMSDs/Å values between calculated and experimental structures during MD trajectories.

Protein	200 ns	2000 ns
Chignolin	3.982	4.123
CLN025	4.180	4.186
2I9M	3.834	2.902
Trp-cage	5.007	1.546
FSD-1	8.313	7.460
HPH	10.206	12.345
Crambin	8.266	8.843

**Table 4:** The minimum and average RMSDs/Å values in REMD trajectories.

Protein	Min	Average
Chignolin	0.474	4.191
CLN025	0.446	4.694
2I9M	0.582	3.375
Trp-cage	0.485	1.966
FSD-1	2.555	7.697
HPH	0.959	8.769
Crambin	3.898	7.595
